# Influence of Weather Conditions and Mechanical Reclamation on Molding Sand with Alkali-Phenolic Binder for Manganese Cast Steel

**DOI:** 10.3390/ma16010071

**Published:** 2022-12-21

**Authors:** Mariusz Łucarz, Dariusz Drożyński, Aldona Garbacz-Klempka, Jan Jezierski, Dariusz Bartocha, Tomasz Wróbel, Krzysztof Kostrzewa, Edward Feliks

**Affiliations:** 1 Faculty of Foundry Engineering, AGH University of Science and Technology, 30 A. Mickiewicza, 30-059 Kraków, Poland; 2 Huta Małapanew Sp. z o.o., 1 Kolejowa, 46-040 Ozimek, Poland; 3 Department of Foundry Engineering, Silesian University of Technology, 7 Towarowa, 44-100 Gliwice, Poland

**Keywords:** molding sand, sand reclamation, Alphaset, manganese cast steel, steel casting

## Abstract

The article presents the results of research on the properties of molding sands in Alphaset technology (alkaline-phenolic). These sands are often used in steel foundries, producing large castings. However, knowledge about them, and especially about the changeability of their properties with the change of environmental conditions (seasons), is still insufficient. Various compositions of molding sand were analyzed based on fresh chromite sand and reclaimed sand. A binder and hardener in various mass ratios were used to prepare the mass. The research methodology included, among others, tests of tensile and bending strength, permeability, abrasion, gas emissivity, and ignition losses. These tests were carried out for summer and winter conditions. The results showed the optimal proportions of resin and hardener, showed the influence of ambient temperature on the properties of the molding sand, and the possible ratio of reclaimed sand in relation to fresh sand. However, you should always remember to verify them under the conditions of a specific foundry.

## 1. Introduction

The alkaline-phenolic process (the so-called Alphaset), in addition to being used for the production of small- and medium-size castings, can be successfully used for large-size steel castings [1], such as the so-called railroad stems (frogs) and turnouts. It can be used for any casting alloy, but for alloys with a higher melting point, such as manganese cast steels, a more temperature-resistant grain matrix is required. This requirement is met by chromite sand. It is a material that contains mainly FeCr_2_O_4_ chromite (but also FeO and Cr_2_O_3_). Theoretically, chromite contains 67.91% Cr_2_O_3_. It has a black, slightly grayish color, density 4500–4800 kg/m^3^, Mohs hardness 5.5. It is also a brittle material, hence its angular shape. The melting point is about 2180 °C, the sintering temperature is 1350–1500 °C, pH 7–10 [2]. Chromite sand prevents liquid alloy from penetrating into the molding sand better than silica sand. Therefore, coarser chromite sand can be used without deteriorating the surface quality of the castings. It also has the advantage for the production of railway track components because it is less capable of reacting with metal oxides and has poor wettability. Therefore, it can be used successfully for the preparation of sands intended for the production of castings of austenitic manganese cast steel [2]. Depending on the resin manufacturer, 1 to 2 parts by weight of a high-alkaline phenolic resin (resol type) are added to the grain matrix in the alkaline-phenolic process, followed by an aliphatic ester in a ratio of 18 to 25% with respect to the resin. When using chromite or olivine sand, more resin is added, i.e., about 2 parts by weight and 20% by weight of esters (with a resin to hardener ratio of 5/1). Chemical reactions between certain esters or their mixtures with a resin containing potassium hydroxide lead to hardening and solidification. Depending on the ester used, the hardening time can be regulated, usually up to 5–30 min [3,4,5,6]. The alkaline-phenolic process is a binary binder system consisting of an aqueous reactor and the liquid ester as the reaction partner. For the production of molding sand in this process, alkali-phenolic resins hardened with an organic ester (based on alcohol) are used. The phenol resins used in the system are prepared by reacting a 30–55% formaldehyde solution with a smaller amount of phenol, and using a strong alkaline catalyst, for example, sodium or potassium hydroxide, at a temperature below 110 °C (pH > 7) [7]. A huge selection of hardeners allows the production of a variety of mixtures with different curing times, which is one of the most important parameters of resin-bonded molding sand. The most important advantages of the Alphaset system (also called ester-hardened alkali-phenolic no-bake binder system) are the following: low odor, practically no smoke, easy-to-apply coatings, good surface quality of castings, low tendency to form veins, minimal erosion, and very good hot strength [7]. It is a resin dedicated to cast steel, and as both the literature and the experience of many foundries indicate, its use improves the quality of castings, thus improving the foundry’s economic indicators. In addition, ecological reasons are very important in this case, which is very important in the countries of the European Union. Therefore, steel foundries in Poland and elsewhere are successively switching to this type of sand.

The main attribute of the alkali-phenolic process is that a two-step hardening process takes place during the formation of chemical bonds. At ambient temperatures, an initial (partial) setting (polymerization of the binder) takes place, which provides the molding sand or core with sufficient handling strength, ensuring the thermoplasticity of the prepared sand at medium temperatures. This thermoplasticity compensates for the thermal expansion of the sand grains, which in turn almost completely eliminates cracks in the casting molds and melt-leakage problems. The heat generated by the liquid metal (casting) completes the solidification of the resin. Therefore, this technology guarantees dimensional stability and resistance to liquid metal erosion [8,9].

The alkaline-phenolic system is commonly used in foreign foundry plants, while in Poland it is not yet so popular. For example, it has been approved as an industry standard for the production of steel castings in England [10]. All the advantages mentioned above indicate that this technology is the most appropriate and environmentally friendly among those proposed for the foundry industry. Its advantages are most noticeable in steel foundries, as it significantly improves the quality of castings and reduces the costs associated with their processing. Furthermore, the total amount of hazardous substances (mainly gases) generated by using this bonding system is much lower than that of other resin-based systems [11]. Further experiments on gas emissions by commercial Alphaset binders showed the same result: Their quality from this point of view is better than in the case of other competing substances, but they differ significantly depending on the suppliers [12,13]. However, in this system, there is a significant problem with the reclamation process of the used molding sand, which means that specific methods must be used to ensure that the reused sand meets quality requirements [14]. There are few scientific articles comparing the advantages and disadvantages of the alkaline-phenolic and furan processes. In one of them, it was found that the choice between two binders (alkaline-phenolic and furan) for green sand foundries depends on many factors, the main of which are: the type of alloy being cast, compatibility with cheap and available sand, and investment opportunities for mechanical and thermal reclamation. Moreover, in the same article, the authors claim that modern Alphaset and furan systems are able to work as pure bonding systems with an optimal level of reclaim addition, while still meeting the functional requirements of modern foundries [14].

In terms of reclamation, a two-step process is essential for the best results. It should consist of good quality mechanical reclamation followed by a thermal one for best results. After the first stage of reclamation, the efficiency can reach 60–80%, but it is crucial to use sodium resin to bind this matrix to the reclaimed sand. After thermal reclamation, fresh sand can be almost 100% recycled material. However, to make it possible, special additives (0.6–1.0%) should be applied to vibratory regenerated sand just before its introduction into the thermal process section [7]. Another important issue is the management of post-recycling dust, which the authors have already discussed in [15]. They analyzed several molding sand compositions using a variety of binder reagents, including those used in the Alphaset process. The results confirmed their usefulness in modern reclamation processes. The authors explained the importance of reclamation and waste management in their paper on the approach to the problem of foundry waste [16]. They presented the vast experience of their foundry industry and focused on the reuse of solid waste, including the used sand matrix. In [17], the authors present the results of research on the mechanical properties of molding sand bound with Alphaset resin, which demonstrates its good quality. The authors [1] present an analysis of various bonding systems in terms of large-size castings, stating that the development of optimal molding sands for casting production, taking into account restrictive environmental protection requirements, is a very topical issue from the point of view of the structure of the European foundry industry. They claim that the strength properties of Alphaset are good enough to be attractive to foundries [1].

This article was created on the basis of a review of the literature on the subject and on the basis of the authors’ scientific and practical experience in the conditions of many foundries, including steel foundries. Weather conditions, as well as the method and effectiveness of mechanical reclamation, were found to have a significant impact on the properties of the molding sand and thus on the quality of the castings produced, which required the conduct of dedicated research described in the article.

## 2. Materials and Methods

The sands prepared in the alkaline-phenolic process were tested with Permabind 440 binder and Permabind P8 hardener. One of the hardeners was used with full awareness for the tests, because in the case of molding sand preparation in laboratory conditions, the sample preparation time is very short, so the durability of the sand is not important in this case. On the other hand, the manufacturer offers an appropriate range of ester catalysts with various options for curing time that meet the needs of most foundries. The matrix of all the sands was chromite sand and mechanical reclaim obtained from the used mass:

- chromite sand (P100) with the following parameters: medium sand (0.20/0.32/0.40), medium grain size d_L_ = 0.29 mm, homogeneous (J87) with a specific surface area of 4.24 m^2^/kg,
- used molding sand (R100) with the following parameters: (0.20/0.40/0.32), with an average grain size d_L_ = 0.29 mm, homogeneity (J83) with a specific surface area of 5.72 m^2^/kg,
- mechanical reclaim (RM15M100) with the following parameters: (0.20/0.40/0.32), with an average grain size d_L_ = 0.23 mm, homogeneity (J75) with a specific surface area of 4.33 m^2^/kg.

Table 1 shows the compositions of the molding sands used during the experiments.

The designations in brackets refer to the same composition of the molding sand, but the tests were carried out under the assumed conditions: spring-autumn (S-A) and summer (S). This article does not recommend any system or suggest the best one. The choice is always a matter of an optimization process based on several factors, including the specific conditions of the particular foundry. In the case of this publication, it is only a demonstration of the phenomena that occur in the production process.

Individual compositions of the molding sand were prepared in a laboratory blade mixer Vogel & Schemmann Maschinen GmbH, type Labor Mischka 00GF/79. First, a weighed portion of the grain matrix was poured into the mixer, into which the hardener was dosed and mixed for 45 s, then the resin was dosed and mixed for another 45 s. The molds were mounted on the LUZ-1 type vibration compaction apparatus, manufactured by WADAP Wadowice, which was turned on for a period of 15 s using a vibration amplitude of 90%. The bulk density (apparent) for chromite sand is approximately 2.55–2.65 g/cm^3^. After compaction, the top was removed, the excess mass was cut off and the main part of the mold shaping the samples was disassembled. The prepared shaped samples were left to harden under ambient conditions. Eight-shape specimens were produced to determine the tensile strength $R_{m}^{u}$, longitudinal specimens to test the bending strength $R_{g}^{u},$ and cylindrical specimens for determining the permeability $P^{u}$ and abrasion S were produced.

The tensile strength $R_{m}^{u}$ and bending strength $R_{g}^{u}$ of the hardened samples were determined according to the PN-83/H-11073 standard. Measurements were carried out on a universal apparatus to determine the strength type LRu-2e by Multiserw-Morek after three hardening times (compacted samples left for setting): 1, 3, and 24 h. At each curing time, the strength was measured on three pieces, and the results presented in the graphs are the arithmetic mean of these measurements.

The permeability of $P^{u}$ in the hardened state was determined according to the PN-80/H-11072 standard. Measurements were made using a fast method on the LPiR-1 apparatus manufactured by WADAP Wadowice. The hardened cylindrical sample was fixed in a special sleeve with an inflatable rubber gasket inside, pressed against the side surface of the sample with compressed air using a hand pump. The permeability for each molding sand was determined on three pieces cured for 24 h, and the results presented in the graph are the arithmetic mean of these measurements.

The abrasiveness of the tested sands was determined according to the BN-77/4024-02 standard. Measurements were made on a device manufactured by Huta Stalowa Wola. The measurement consisted of mounting a weighed cylindrical sample (hardened for 24 h), made of the tested sand, in the apparatus holder. The piece was then rotated at a speed of 1 rps with an electric motor. During the experiment, the shot falls from the height of 307 mm on the rotating sample and causes its abrasion. 1750 g of steel shot were used, with a diameter of 1 mm, weighed with an accuracy of 1.0 g. The measurement was carried out on three samples and the results presented in the graph are the arithmetic mean.

The experiments were carried out in two seasons of the year, in spring, when the ambient temperature ranged from 10.8–11.7 °C, and the relative humidity varied within the range of 37.3–43.4%, and in summer, when the ambient temperature was recorded in the range 27.7–28.9 °C, and relative humidity 35.2–38.6%. Since in the spring weather conditions in Central Europe (Poland) are similar to autumn weather conditions, the designations spring-autumn—S-A and summer—S have been introduced in the graphs for individual seasons.

For the purposes of the analysis of gas emission, a sample of the binder was prepared by mixing the resin with the hardener in the following ratio: per 1 weight part of the resin, 25% of the hardener in relation to the resin. After 24 h, the cured binder was crushed and prepared for gas emission tests. Gas emission measurements of the samples were carried out on a stand equipped with a pipe furnace type PRC 30M/1300 by CZYLOK, a peristaltic pump type BT100-2J by LongerPump and a control and recording unit [18,19,20].

The measurement consisted of heating a tubular furnace (quartz tube) to a temperature of 1000 °C, into which a ceramic boat-shaped mold with a sample of the tested material weighing 0.2 g (resin) was then introduced (outside the heating zone), and the used sand weighing 1 or 3 g (weighed to within ±0.001 g). The measurements results were converted to one gram of sample weight. The curves presented in the figures were created as the average of three determinations of gas emission. Sand samples subjected to heating were obtained from samples for strength tests. After the analyzed sample of material was introduced, the pipe was tightly closed on one side and connected to a peristaltic pump on the other side, to create a vacuum in the reactor and pump out the generated gases. The sample was then introduced into the heating zone, where it was heated very quickly to the measurement temperature, and the volume of gas generated was recorded.

The measurement of ignition losses was carried out on samples of crushed sand from samples for strength tests. The tests were carried out at a temperature of 950 °C in a SNOL 8.2/1100 muffle furnace on two 30 g samples weighed in quartz crucibles. The time to heat the samples was 2 h.

To verify the change in the weight of the chromite sand matrix, a thermal analysis test was performed using a TA Instruments SDT Q600 Thermal Analyzer (DSC/TGA) The furnace was heated at a rate of 10 °C/min to 1000 °C, greater than the loss in the ignition measurement.

Microstructure studies were carried out using a Hitachi S-3400N scanning electron microscope (SEM), where the source of electrons was a tungsten cannon with thermal emission. Observation of the surface of the reclaimed sand was performed in backscatter electron contrast (BSE). The variable vacuum VP-SEM mode (20 Pa) was used.

**Figure 1:**
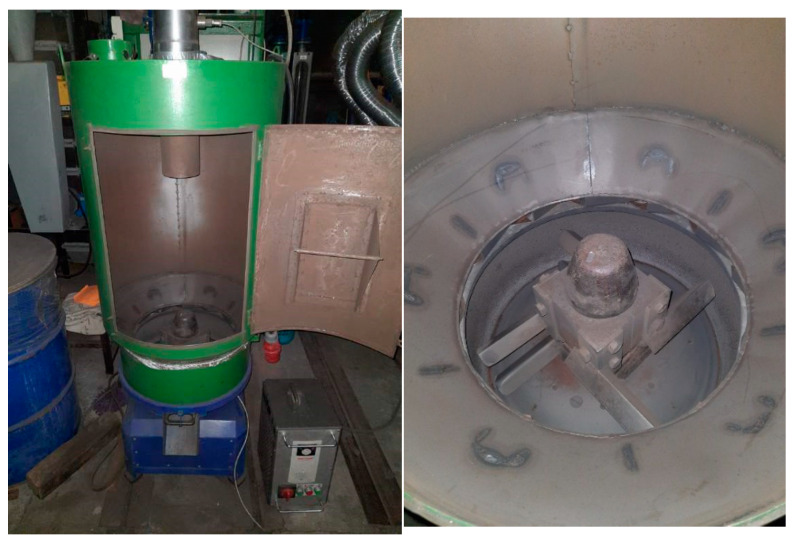
View of the experimental RD-6 rotor regenerator equipped with a dust extraction system generated in the matrix abrasive and crushing process.

The reclaimed sands marked with the symbols RM5M100, RM10M100, and RM15M100 were obtained as a result of the reclamation of the grain matrix (used sand—R100) on the mechanical rotor regenerator RD-6 (Figure 1), performing the reclamation process in 6 kg portions of charge, for 5 min, 10 min and 15 min, with the rotating speed of the working elements 1150 rpm.

## 3. Results and Discussion

**Figure 2:**
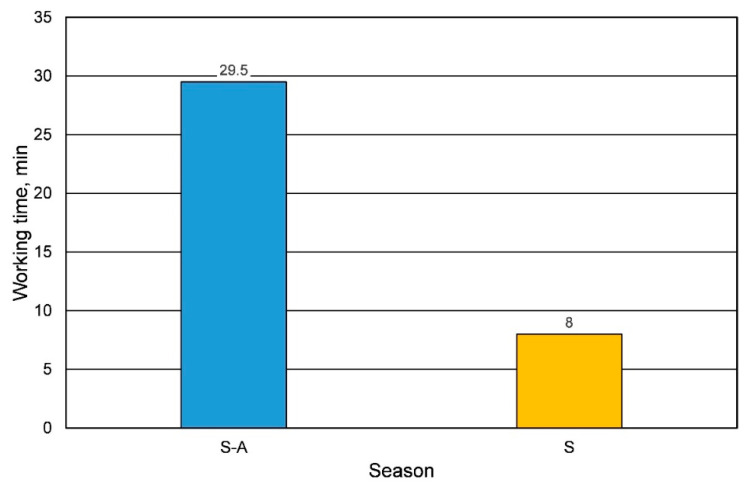
Lifetime of the molding sand, depending on the season.

The production process of casting molds based on chemically set binders depends on weather conditions. In the article, tests were carried out on the effect of temperature on the technological parameters of the sand, depending on the amount of resin added to the molding sand and the constant amount of hardener dosed. As mentioned earlier, the same catalyst was used in different seasons of the year. In each of the periods analyzed, the sand life was determined as the basic parameter of the casting mold preparation. The results are shown in Figure 2. The analyzed parameter was determined for the composition P100Z1.0. For other compositions (the amount of resin added), a similar lifetime was obtained (a difference of about 1 min) in certain seasons of the year.

**Figure 3:**
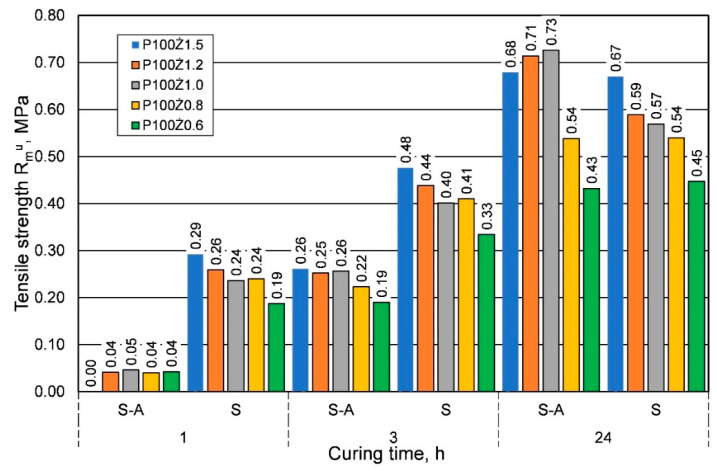
Tensile strength $R_{m}^{u}$ of molding sand made on the basis of fresh chromite sand, depending on weather conditions, for different amounts of added binder.

Figure 3 compares the results of the effect of the amount of resin on the tensile strength $R_{m}^{u}$ with the addition of the same amount of hardener.

**Figure 4:**
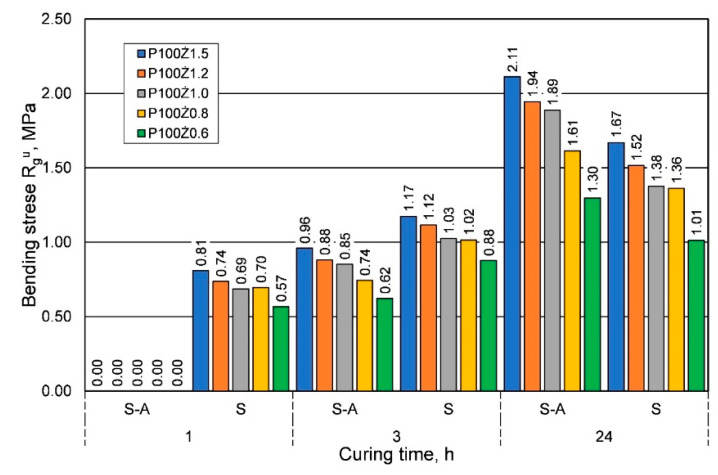
Bending strength $R_{g}^{u}$ of molding sand based on fresh chromite sand, depending on weather conditions, for different amounts of binder added.

The comparison presented in Figure 3 shows that for the summer weather conditions, for shorter curing times, a greater strength was obtained, regardless of the amount of resin added. On the other hand, for the longest curing time of 24 h, lower or comparable tensile strengths were obtained. Figure 4 shows a comparison of the results of the influence of ambient temperature (seasons) on the bending strength $R_{g}^{u}$.

**Figure 5:**
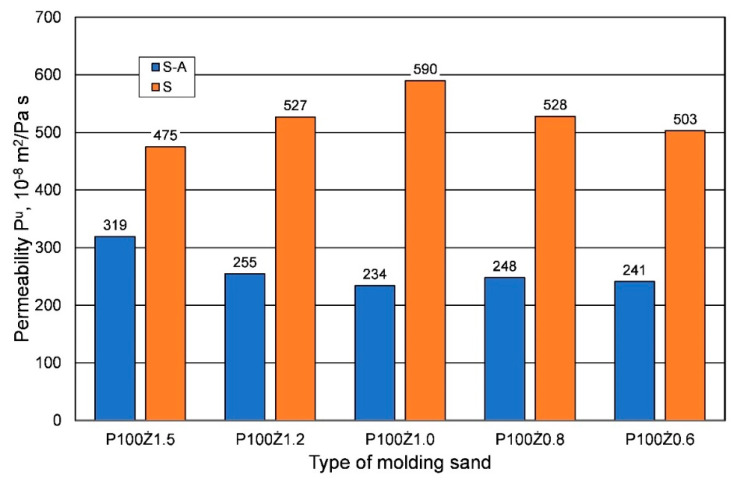
Permeability $P^{u}$ of hardened molding sand (24 h) prepared on the basis of fresh chromite sand, depending on weather conditions, for different amounts of added binder.

For bending strength, a similar effect of weather conditions was observed as for tensile strength; however, for the longest curing time, significantly lower bending strengths were obtained in the summer. In the analyzed case, it is worth noting that, regardless of the weather conditions, the lower the resin content in the molding sand, the lower the bending strength. An important parameter of the molding sand when manufacturing large-size castings from alloys with a high melting point is its permeability $P^{u}$. Figure 5 shows the results obtained from the analyzed parameter of hardened sand molding (after 24 h) depending on the binder content, but also on the weather conditions. Research shows that for the same sand matrix, the same resin, and the same amount of hardener, sands prepared under summer conditions have better permeability. More than twice the permeability was obtained for most of the resin/hardener ratios.

**Figure 6:**
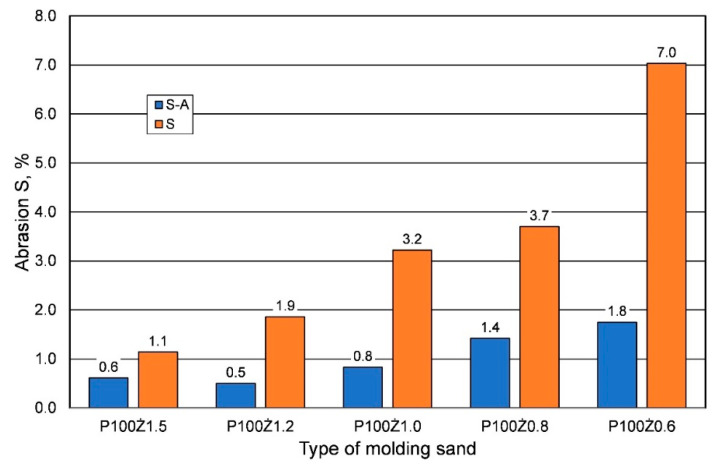
Abrasion S of the hardened sand (after 24 h) prepared on the basis of fresh chromite sand, depending on weather conditions, for different amounts of added binder.

Another important parameter of the molding sand is its abrasion resistance S. The results of this parameter measurement, depending on the weather conditions, determined after 24 h of hardening, are shown in Figure 6. As the strength parameters of the sands prepared in summer conditions decreased for the curing time of 24 h, this resulted in an increase in the abrasion of the molding sand. The less resin there was in the molding sand, the higher the abrasion resistance, regardless of the weather conditions.

**Figure 7:**
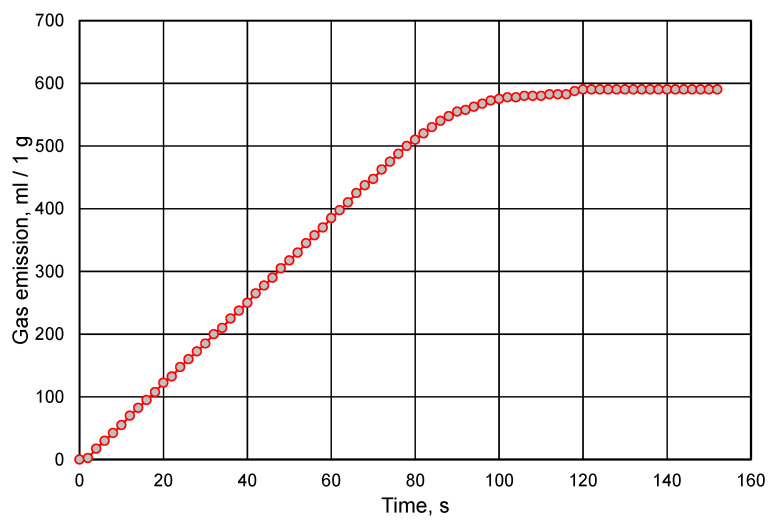
Measurement of Permabind resin gas emission.

**Figure 8:**
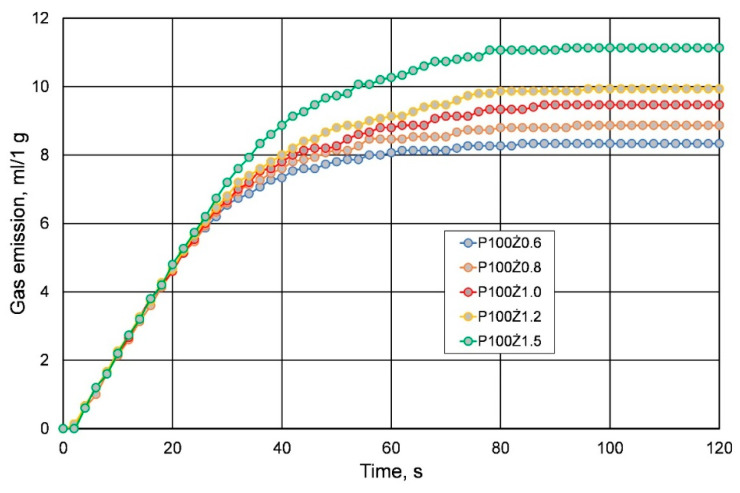
Measurement of gas emission of molding sands with different resin content.

When looking for the optimal composition of the molding sand, with regard to the amount of resin and hardener, one should follow the rule that the less organic binder in the sand, the better. The high content of organic compounds in the molding sand is degraded or destroyed as a result of contact with liquid metal, causing the formation of gases in the mold cavity, which in turn affects the quality of the castings [21,22]. Therefore, an analysis of the amount of gases emitted was carried out. Figure 7 shows an exemplary analysis of gas emissions from the bound binder with a share of 25% of the hardener in relation to the resin. This result is a reference point for the emission of gases from the tested molding sands. When resin and hardener constitute a small amount in the composition of molding sand, the amounts of released gases are lower. However, the amount of resin added to the prepared sand is significant, which is shown in Figure 8. The data presented in Figure 8 refer to the molding sands prepared in the summer.

**Figure 9:**
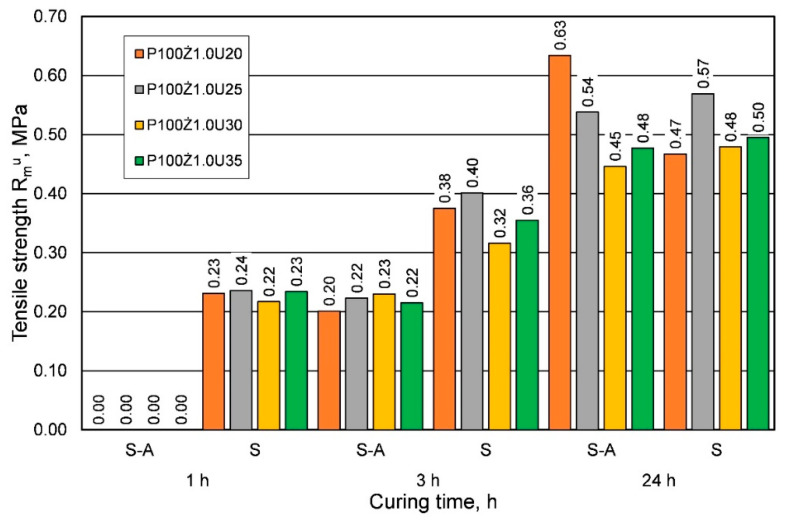
Tensile strength $R_{m}^{u}$ of molding sand based on fresh chromite sand, depending on weather conditions, for different amounts of hardener added.

**Figure 10:**
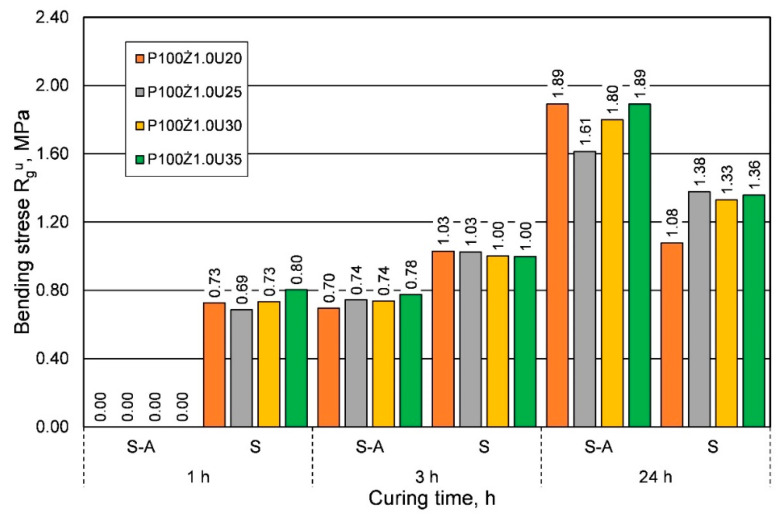
Bending strength $R_{g}^{u}$ of molding sand based on fresh chromite sand, depending on weather conditions, for different amounts of added hardener.

When comparing all parameters analyzed, to determine the best composition of the molding sand, from the point of view of weather conditions, sand quality, cost-effectiveness of the process and ecological aspect, it was found that the best is the molding sand with 100 weight parts of the grain matrix (in this case chromite matrix), 1.0–1.2% resin and 25% hardener in relation to the resin. The next step was to determine the effect of the amount of hardener added to the constant amount of resin dosed to the grain matrix. Figure 9 shows the results of the tensile strength tests, and Figure 10 shows the bending strength.

**Figure 11:**
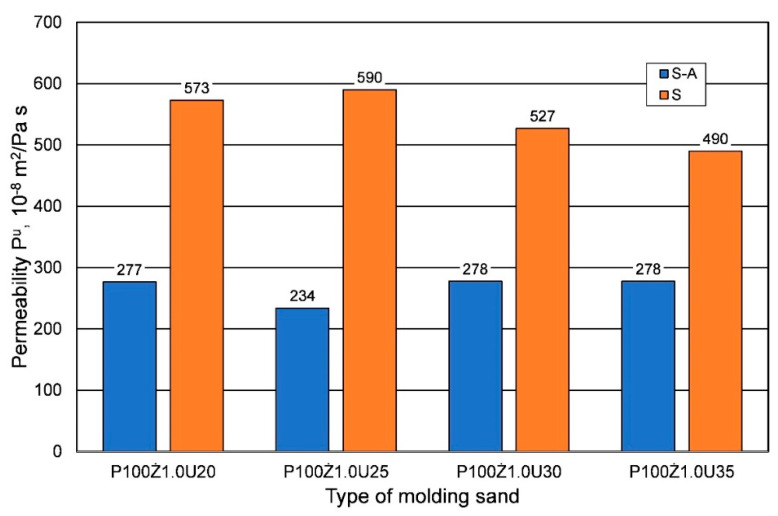
Permeability $P^{u}$ of hardened molding sand (24 h) prepared on the basis of fresh chromite sand, depending on weather conditions, for different amounts of hardener added.

The results obtained confirm the influence of ambient temperature on the values of individual strength properties, while the amount of hardener added does not clearly affect their change. For the curing time of 24 h, a decrease in strength is visible at the summer temperature. As part of the investigation, it was also determined how the setting temperature and the amount of added hardener affect the permeability of the mass in the hardened state of Pu. Figure 11 presents the results obtained.

**Figure 12:**
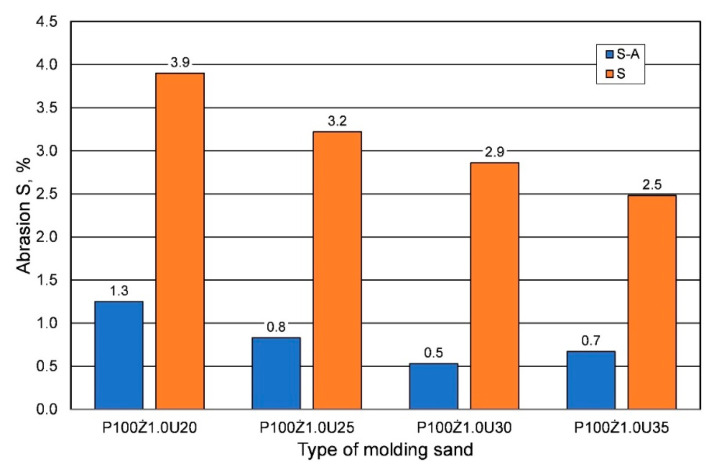
Abrasion S of hardened molding sand (after 24 h) prepared on the basis of fresh chromite sand, depending on weather conditions, for different amounts of added hardener.

The results obtained indicate a better permeability of the molding sands set under summer conditions, but without a clear influence of the amount of hardener dosed on the examined parameter for both temperature ranges. The tests carried out also determined the abrasiveness of the molding sand for the change in hardener dose to the sand. Figure 12 shows the results obtained. In this case, a higher sand binding temperature resulted in a higher abrasion. You can also see a slight reduction in abrasion with increasing the amount of hardener dosed.

**Figure 13:**
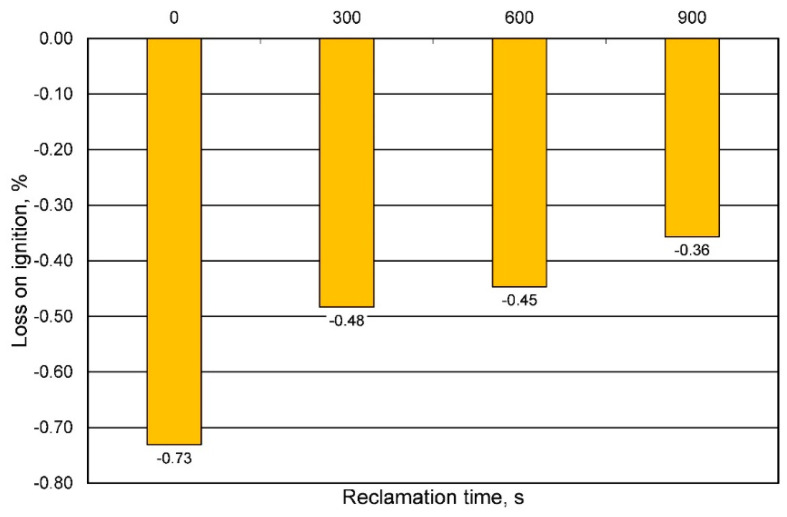
Ignition losses of used sand and reclaimed sand for different times of mechanical reclamation.

Chromite sand is an expensive material in the production of foundry molds. Therefore, its proper management is of great importance. Extending its useful life in the production process requires the use of a reclamation process. The grain matrix recovery process is in line with the current trend related to the so-called circular economy. Reclamation of used molding sand with organic binders can be performed using a mechanical or thermal method. Due to the widespread assessment that the thermal process is costly, the matrix recovery attempt was carried out using a mechanical method. To select the appropriate intensity of interactions, the process was carried out for 3 times: 300 s, 600 s, and 900 s. The quality of the reclaims obtained was assessed by determining the ignition loss. Figure 13 shows the results obtained for various periods of molding sand processing.

**Figure 14:**
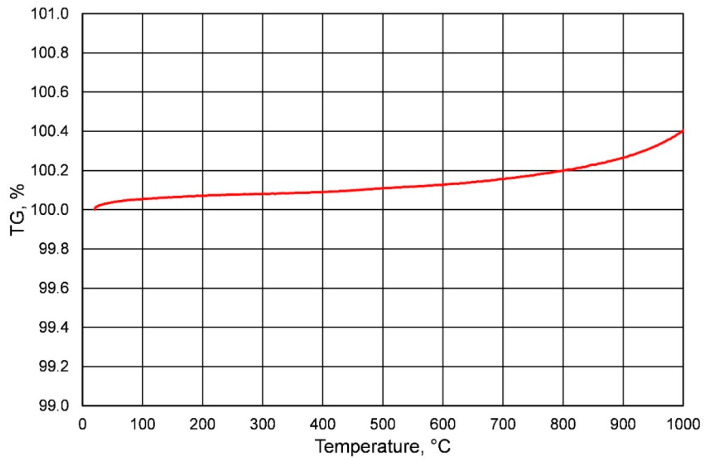
Thermal analysis of the P100 sand matrix.

For quartz-based molding sand with an organic binder, along with the duration of mechanical reclamation, ignition losses decrease (abrasion of the binder used) [23] and have a positive value. In the analyzed case, as a result of roasting, an increase in mass was found, which in turn meant that the losses on ignition obtained for the molding sand on the chromite matrix had a negative value. This phenomenon can be explained by the mass change (increase) of the chromite matrix as a result of the temperature. Figure 14 shows the result of the thermal analysis of the fresh P100 chromite matrix, where the mass of chromite sand increases with increasing temperature.

**Figure 15:**
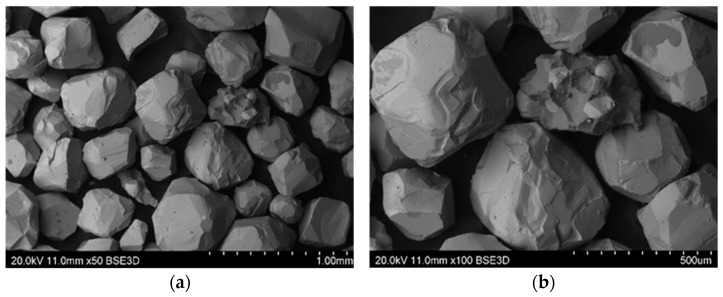
BSD images from the scanning electron microscope of the P100 chromite grain matrix: (**a**) mag. 50×, (**b**) mag. 100×.

**Figure 16:**
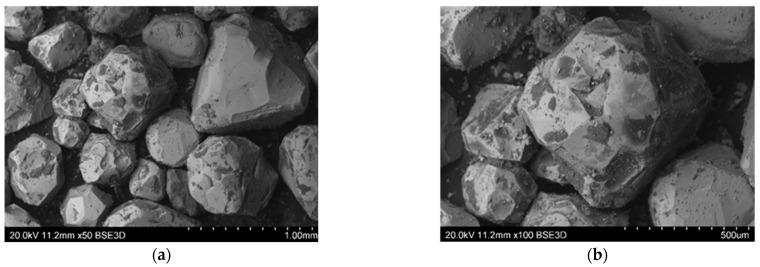
BSD images from the scanning electron microscope of the used sand on the R100 chromite matrix: (**a**) mag. 50×, (**b**) mag. 100×.

**Figure 17:**
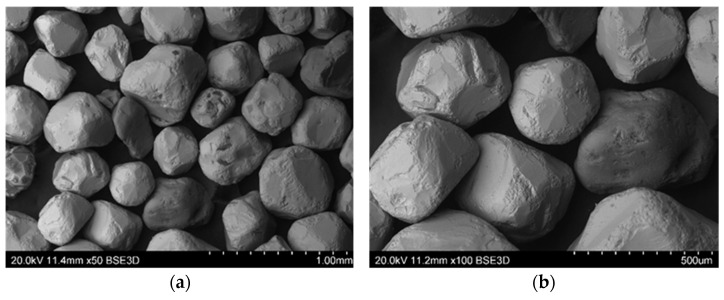
BSD images from the scanning electron microscope of mechanical reclaimed material after 5 min of treatment of the used sand on the RM5M100 chromite matrix: (**a**) mag. 50×, (**b**) mag 100×.

**Figure 18:**
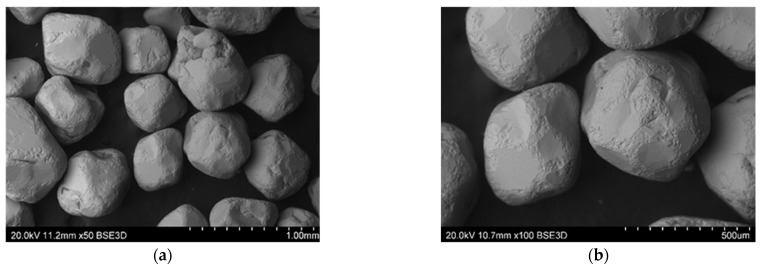
BSD images from the scanning electron microscope of mechanical reclaimed material after 15 min of treatment of the used sand on the RM10M100 chromite matrix: (**a**) mag. 50×, (**b**) mag. 100×.

**Figure 19:**
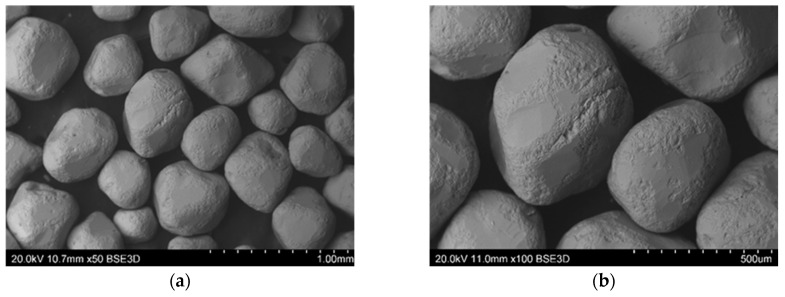
BSD images from the scanning electron microscope of the mechanical reclaimed material after 15 min of treatment of the used sand on the RM15M100 chromite matrix: (**a**) mag. 50×, (**b**) mag. 100×.

Mechanical regeneration of sands with organic binders cannot remove the entire amount of binder, especially the one accumulated in cavities and surface irregularities of matrix grains. Therefore, despite increasing the regeneration time, satisfactory cleaning of the grain matrix was not achieved. On the other hand, increasing the regeneration time leads to a decrease in the grain matrix yield by increasing the abrasion of its surface and rounding of the matrix grains (especially angular chromite sand), which can be seen in the images presented from the scanning microscope (Figure 15, Figure 16, Figure 17, Figure 18 and Figure 19). When the scan photos of the fresh grain matrix, the used sand, and mechanical regenerates are compared, a change in the surface is clearly visible: its shape and the degree of coverage with the bound binder. With an increase in the time of mechanical regeneration, the grains are more “clean” and the edges of the grains are more rounded.

**Figure 20:**
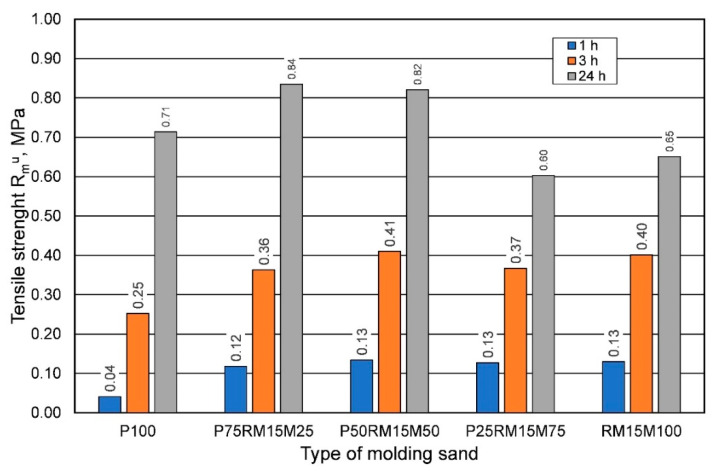
Tensile strength $R_{m}^{u}$ of the molding sand depending on the sand matrix used.

Taking into account the economic as well as the ecological aspect, the next step of the research was to determine the technological parameters of the molding sand with the use of reclaimed sand after 900 s of mechanical treatment (with the lowest loss of ignition, preferably cleaned of the used binder), in specific proportions to fresh sand. Research in this area was carried out in the spring. Figure 20 presents the results of tensile strength tests of the molding sand made on the basis of sand matrix, prepared with fresh sand and recovered mechanical reclaim in various proportions. Based on the results obtained, it was found that the addition of 25% and 50% did not worsen the tensile strength of the sand, but actually improved it.

**Figure 21:**
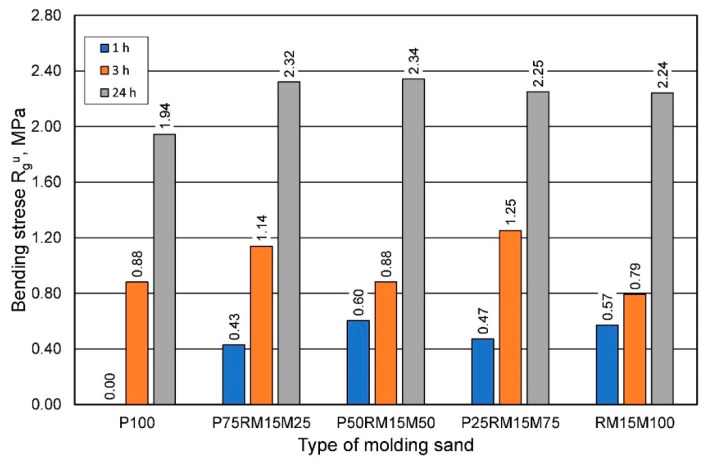
The bending strength $R_{g}^{u}$ of the molding sand depends on the grain matrix used.

A similar dependence can be seen for the bending strength, with higher strengths obtained for the greater share of reclaimed sand in the molding sand than for sand prepared on the fresh matrix (Figure 21).

**Figure 22:**
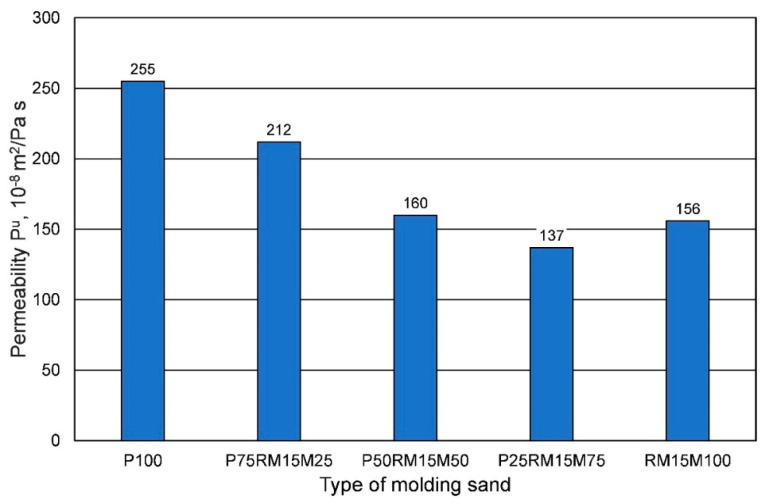
Permeability $P^{u}$ of the molding sand, depending on the grain matrix used.

Increasing the share of mechanical reclaim in fresh chromite sand adversely affects the permeability of the molding sand, as shown in Figure 22.

**Figure 23:**
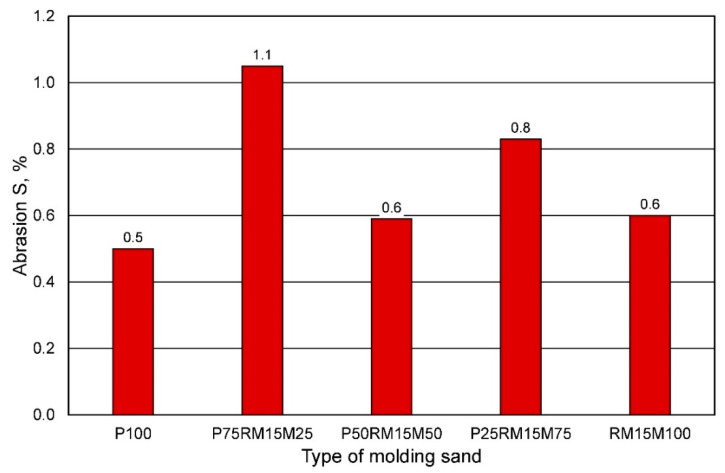
Abrasion S of the molding sand, depending on the sand matrix used.

However, the abrasion resistance of the tested molding sands made on the basis of various compositions of grain matrices does not show a clear tendency, although the addition of reclaimed material slightly increases the tested parameter (Figure 23).

## 4. Summary and Conclusions

The article presents the results of tests on the molding sand with an alkali-phenolic binder based on fresh chromite sand and the mechanically reclaimed matrix. Reclaim was obtained as a result of the mechanical abrasion process performed on the used molding sand on a chromite matrix. The first part of the research was to determine the impact of atmospheric conditions on the technological parameters of the sand. The next step was to determine the influence of the proportion of added components on the mechanical properties and other technological parameters of the tested molding sands.

As expected, a higher temperature accelerates the bonding reactions, and hence the strengths of both $R_{m}^{u}$ and $R_{g}^{u}$, for shorter sample setting times, are higher. However, for the longest time after which the samples were tested, a decrease in strength is visible. This state may result from greater reactivity (more intense and fully realized binding reaction), which leads to the release of more volatile substances (this is due to time), which ultimately results in weakening of the bonds and a decrease in the tested strengths. This regularity is visible regardless of the amount of resin used to prepare the sand. From a scientific point of view, this aspect requires further in-depth analysis.

The opinion presented above is confirmed by permeability tests performed after 24 h. In summer conditions, the value of this parameter clearly increases, reaching the optimum value for the resin content of 1.0%, which may be the result of the creation of larger intergrain spaces and better gas migration in the porous structure of the molding sand. The weakening of the bonds, resulting from the increased reactivity of the resin at higher temperatures, is also noticeable in the abrasiveness value of the molding sand. The less binder in the sand and the higher the temperature, the greater the abrasiveness.

Taking into account all the results of the completed scope of research, looking for an optimal solution (taking into account the economy of the process, manufacturability from the point of view of the occurring phenomena and obtained parameters, ecology), it seems that the addition of 1 to 1.2% resin to the fresh grain matrix is a compromise approach. The high density of chromite sand requires the addition of 30 ± 50% less binding material than quartz sand, but a high degree of grains angularity can reduce this difference. This may explain that with a constant amount of resin and hardener dosed to the matrix with an increasing share of reclaimed material (more rounded grains), higher strengths of the molding sand were obtained than for the fresh chromite matrix. Of course, this effect works only to a certain extent, therefore, the suggested amount of regenerate added to the near-pattern sand is recommended in the range of 50 ± 75%. It should be remembered that mechanically reclaimed material contains bound resin, which in contact with liquid metal will burn out and increase gas emissions, which may affect the quality of castings. However, as laboratory tests have shown, in the case of mold filling sand, the amount of reclaim added to the sand can exceed 75%. It should be recalled here that proper hardening of the resin takes place only after contact with the liquid casting alloy (two-stage bonding process). The amount of reclaim added to the molding sand can be assessed only in real conditions, taking into account many variables (e.g., degree of resin burnout resulting from the amount of heat supplied from the liquid metal, i.e., casting weight), which may affect the quality of the prepared casting mold.

## Figures and Tables

**Table 1:** Composition of molding sands with an alkaline-phenolic binder.

Sand Label	Chromite Sand [wt.]	Mechanically Reclaimed Sand [wt.]	Resin [wt.]	Hardener [% Ratio to the Resin]
P100Ż1.5 (S-A, S)	100		1.5	25
P100Ż1.2 (S-A, S)	100		1.2	25
P100Ż1.0 (S-A, S)	100		1.0	25
P100Ż0.8 (S-A, S)	100		0.8	25
P100Ż0.6 (S-A, S)	100		0.6	25
P100Ż1.0U20 (S-A, S)	100		1.0	20
P100Ż1.0U25 (S-A, S)	100		1.0	25
P100Ż1.0U30 (S-A, S)	100		1.0	30
P100Ż1.0U35 (S-A, S)	100		1.0	35
P100	100	0	1.2	25
P75RM15M25	75	25	1.2	25
P50RM15M50	50	50	1.2	25
P25RM15M75	25	75	1.2	25
RM15M100	0	100	1.2	25

## Data Availability

Not applicable.
